# Five years of Hospital at Home adoption in Catalonia: impact, challenges, and proposals for quality assurance

**DOI:** 10.1186/s12913-024-10603-1

**Published:** 2024-02-01

**Authors:** Rubèn González-Colom, Gerard Carot-Sans, Emili Vela, Mireia Espallargues, Carme Hernández, Francesc Xavier Jiménez, David Nicolás, Montserrat Suárez, Elvira Torné, Eulalia Villegas-Bruguera, Fernando Ozores, Isaac Cano, Jordi Piera-Jiménez, Josep Roca

**Affiliations:** 1 Hospital Clínic de Barcelona, Institut d’Investigacions Biomèdiques August Pi I Sunyer (IDIBAPS), Universitat de Barcelona, C/ Villarroel, 170, 08036 Barcelona, Spain; 2 Catalan Health Service, Barcelona, Spain; 3 Digitalization for the Sustainability of the Healthcare (DS3) – IDIBELL, Barcelona, Spain; 4 Agència de Qualitat I Avaluació Sanitàries de Catalunya (AQuAS), Barcelona, Spain; 5 Research Network on Chronicity, Primary Care and Health Promotion (RICAPPS), Madrid, Spain; 6 Hospital Vall d’Hebron, Barcelona, Spain; 7 Hospital Dos de MaigConsorci Sanitari Integral, Barcelona, Spain; 8 Faculty of Informatics, Telecommunications and Multimedia, Universitat Oberta de Catalunya, Barcelona, Spain

**Keywords:** Hospital at home, Implementation science, Integrated care, Key performance indicators, Multimorbidity

## Abstract

**Background:**

Hospital at home (HaH) was increasingly implemented in Catalonia (7.7 M citizens, Spain) achieving regional adoption within the 2011-2015 Health Plan. This study aimed to assess population-wide HaH outcomes over five years (2015-2019) in a consolidated regional program and provide context-independent recommendations for continuous quality improvement of the service.

**Methods:**

A mixed-methods approach was adopted, combining population-based retrospective analyses of registry information with qualitative research. HaH (admission avoidance modality) was compared with a conventional hospitalization group using propensity score matching techniques. We evaluated the 12-month period before the admission, the hospitalization, and use of healthcare resources at 30 days after discharge. A panel of experts discussed the results and provided recommendations for monitoring HaH services.

**Results:**

The adoption of HaH steadily increased from 5,185 episodes/year in 2015 to 8,086 episodes/year in 2019 (total episodes 31,901; mean age 73 (SD 17) years; 79% high-risk patients. Mortality rates were similar between HaH and conventional hospitalization within the episode [76 (0.31%) vs. 112 (0.45%)] and at 30-days after discharge [973(3.94%) vs. 1112(3.24%)]. Likewise, the rates of hospital re-admissions at 30 days after discharge were also similar between groups: 2,00 (8.08%) vs. 1,63 (6.58%)] or ER visits [4,11 (16.62%) vs. 3,97 (16.03%). The 27 hospitals assessed showed high variability in patients’ age, multimorbidity, severity of episodes, recurrences, and length of stay of HaH episodes. Recommendations aiming at enhancing service delivery were produced.

**Conclusions:**

Besides confirming safety and value generation of HaH for selected patients, we found that this service is delivered in a case-mix of different scenarios, encouraging hospital-profiled monitoring of the service.

**Supplementary Information:**

The online version contains supplementary material available at 10.1186/s12913-024-10603-1.

## Background

Two decades after the first report assessing hospital at home (HaH) services [1], which extend acute-level, short term, complex medical care to patients within their homes, this type of care has raised increasing interest as an alternative to inpatient care for selected groups [2–4]. HaH, delivered to entirely substituting the conventional hospitalization, has been associated with several advantages, including patient safety, reduction of nosocomial complications, similar or even better health outcomes compared to conventional hospitalization, high satisfaction levels from both patients and caregivers, and cost savings. In addition, by releasing physical beds, HaH contributes to building capacity for highly specialized care inpatient hospitalization. Moreover, in an integrated care scenario, HaH may become a relevant driver of vertical integration between hospital care and community-based health and social services by enhancing the care continuum.

However, heterogeneities of HaH service profiles are acknowledged, explaining poor comparability among reported experiences [5]. The findings in the literature raise several controversies in different areas, comprising the results of HaH in specific patient groups, modalities of HaH (e.g., admission avoidance or early supported discharge), the most appropriate implementation strategies for HaH services, and the quality-of-care delivery after service adoption [4]. These controversies, and subsequent lack of consensus preclude standardization and continuous quality improvement (CQI) of the service in real-world settings [4]. Therefore, understanding the heterogeneities behind the HaH has become crucial to define service-specific key performance indicators (KPIs) that can be used to ensure quality and sustainability over time and adjust the country-specific regulations of the service.

In Catalonia, a 7.7 million citizens region in North-East Spain with a single public payer (Catalan Health Service) [6, 7], HaH was successfully deployed during the 2011-2015 regional Health Plan [6, 8–10]. The HaH outcomes from that period were used to establish a specific reimbursement scheme based on all patient-refined diagnosis-related groups (APR-DRG) [11, 12] and aimed at consolidating large-scale adoption of HaH services by hospitals across the region [13]. Based on this early experience of HaH implementation at the healthcare system level, Catalonia was selected as Best Practices site for the service in the “Joint Action on implementation of digitally enabled integrated person-centered care” (JADECARE) [14], a program conducted by the European Union, in collaboration with the OECD to promote the assessment and transferability of innovative services with a care continuum approach [15].

With the aim to provide an accurate perspective of the impact of this service and aid future implementers in identifying general, context-independent performance indicators to monitor HaH services, we conducted a mixed-methods study that includes a quantitative retrospective assessment of HaH patients’ characteristics and outcomes, and a co-creation process with a group of experts in HaH.

## Methods

### Overview of study design

The current study combined quantitative and qualitative research methodologies. The quantitative study was a retrospective observational analysis of the characteristics of HaH recipients and health results of hospitalizations occurred between January 1, 2015, and December 31, 2019. To select HaH episodes in the admission avoidance modality, we selected patients with unplanned hospitalizations and less than 24 h between hospital entry and HaH registration. Patients spending more than 24 h in a hospital setting before transitioning home were categorized under the early supported discharge programs and excluded from the analysis. For the qualitative assessment, we conducted focus groups and surveys [16] with a panel of experts in HaH to interpret the results of the quantitative analysis and generate recommendations for CQI.

The quantitative study was reported according to the STROBE [17] guidelines for observational studies, and the qualitative analysis was reported according to the SRQR [18] guidelines.

### Population and data sources

All data used in the quantitative analysis were retrieved from the Catalan Health Surveillance System (CHSS) [19]. Since 2011, the CHSS has collected detailed information on the utilization of healthcare resources by the entire population of Catalonia. The CHSS assembles information on the use of healthcare resources across healthcare tiers, drugs, and other billable healthcare costs, such as non-urgent medical transportation, outpatient rehabilitation, respiratory therapies, and dialysis. We screened the CHSS for all episodes of HaH reported in Catalonia during the study period.

The same database was used to create a retrospectively-matched control group of contemporary conventional hospitalizations. The control group was created using a 1-to-1 propensity score matching (PSM) [20, 21] and Genetic Matching [22] technique based on GENetic Optimization Using Derivatives (GENOUD) [23] algorithm to check and improve covariate balance iteratively. To ensure the comparability of the matched episodes, we screened contemporary admissions within the same hospital with identical Medicare Diagnosis Related Group [12] category. In addition, the patient’s baseline characteristics were characterized and matched using data on demographics (i.e., age and gender), utilization of healthcare resources during the previous year (i.e., hospital admissions, emergency room visits, number of pharmacological prescriptions and the total healthcare expenditure), clinical and social risk factors (i.e., the morbidity burden, using the Adjusted Morbidity Groups [24, 25] (AMG) score, and the presence of active diagnoses related with health-related social needs [26]).

The overall comparability of the matched group was assessed using the Mahalanobis distance [27], and Rubin’s B and Rubin’s R metrics. Comparability after PSM was considered acceptable if Rubin’s B was less than 0.25 and Rubin’s R was between 0.5 and 2 [28].

### Variables and outcomes

The baseline characteristics of study patients (i.e., before admission) included age, sex, morbidity burden measured using the AMG score, hospitalizations, emergency room admissions, and expenditure within the past year. Information regarding the HaH episode included the length of stay (LoS) and the complexity of hospitalization, measured using two case-mix tools: the Case Mix Index- APR-DRG v35 (CMI) [29], broadly used for payment purposes, and the Queralt index [30, 31], recently developed by the Catalan Institute of Health and showing higher performance for predicting general hospitalization endpoints. Readmissions in in-hospital settings and visits to the emergency room without the need to discontinue HaH were also considered clinical outcomes of the HaH episode.

Besides the baseline and episode characteristics, we gathered information regarding healthcare expenditure, hospitalizations, and visits to the emergency room within the 30-days after discharge. Expenditure information was obtained from reimbursements by the Catalan Health Service [32], since no operational costs [33, 34] were available for the entire study group. HaH delivery is reimbursed as a specific healthcare service, with case costs estimated based on the APR-DRG categories of the main diagnostic. Other relevant outcomes included mortality, during the hospitalization and 30 days after discharge.

### Statistical analysis

Before the analysis, we removed from the databases all the incomplete records, duplicate entries, and outliers with unrepresentative baseline characteristics or anomalous LoS with a Z-value greater than |3|.

Since *p-values* tend to drop in large population-based samples, yielding significant differences in most comparisons [35], we used effect size measures to compare the baseline characteristics of the matched HaH individuals with their respective controls to establish the impact of the intervention. Cohen’s D test was used to determine the effect size in numerical variables; the magnitude of the difference was assessed according to the following ranges: weak (< 0.20), small (0.2 – 0.5), moderate (0.5 – 0.8), large (0.8 -1.3), and very large (> 1.3). Cohen’s W test was used for categorical variables, with the following ranges used to assess the magnitude of difference: weak (< 0.10), small (0.1 – 0.3), moderate (0.3 – 0.5), and large (> 50). We computed 1,000 bootstrap replicates in both scenarios to generate the 95% CI.

Categorical variables were summarized as absolute values and percentages, whereas continuous variables were described by the mean and the standard deviation or the median and the interquartile range as appropriate.

To analyze heterogeneity among hospitals regarding the patient profile, we described the age and the morbidity burden (measured using the AMG index) of HaH patients in each center. We also assessed the inclusion bias of each center by measuring the difference in mean age and AMG index between HaH and conventional hospitalizations admitted for the exact cause within the same hospital. Heterogeneity was also assessed regarding the LoS, the complexity of the episode and the repetition rate among HaH patients. In addition to the descriptive analysis, we addressed heterogeneity by conducting an ancillary cluster analysis using the K-means [36] algorithm, incorporating information on the category of the hospital based on the number of hospital beds and their role in their corresponding health district. The average silhouette [37] method was used to determine the optimal number of clusters.

All the data analyses were performed using R [38], version 4.1.1 (R Foundation for Statistical Computing, Vienna, Austria).

### Qualitative assessment

The qualitative study, which followed a grounded theory approach, included two focus group sessions with HaH experts. The first session aimed at interpreting the results obtained in the quantitative analysis described above, whereas the second session sought to discuss the efficiency and value generation of HaH (considering the heterogeneities and challenges of the service) and providing recommendations of core KPIs for CQI of HaH delivery after service adoption. The second session was preceded by the administration of a questionnaire (Supplemental Material) for assessing the consensus strength. Experts were also provided with the 2020 consensus document aiming at regional standardization of HaH [13]. The panel of experts selected the list of core KPIs broadly applicable to HaH services based on the 2020 regional consensus document [13] and the conclusions drawn during focus group sessions.

The panel of 7 experts included 1-to-2 representatives of the most relevant organizations in implementing or assessing HaH services in Catalonia: two members from the Catalan-Balearic Society of Hospital at Home [39], two staff members from the Catalan Health Service [40], one staff member from the Health Quality and Assessment Agency of Catalonia (AQuAS) [41], and two HaH experts from the local JADECARE team. Four out of the seven experts were clinical leaders of different HaH programs. A qualitative research and service design specialist was recruited as a facilitator for planning and leading the expert panel discussions. An extended description of the methodological details is provided in the online Supplemental Material.

## Results

### Adoption and characteristics of hospital at home

**Fig. 1:**
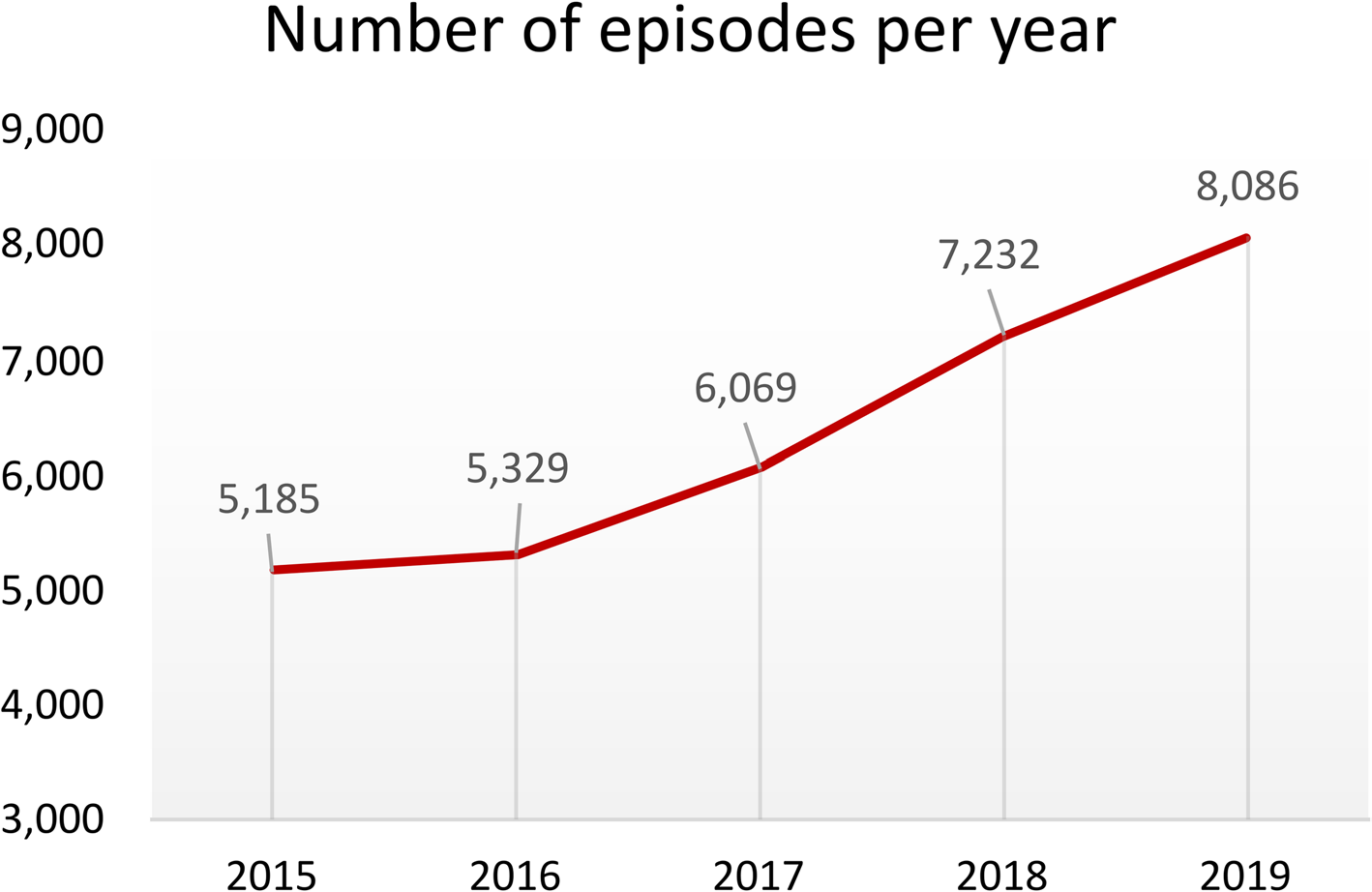
Number of admissions in HaH programs registered in 27 hospitals from Catalonia between 2015–2019

The CHSS registry recorded 31,901 episodes of HaH among the 27 hospitals offering this service to their catchment populations (Fig. 1). Overall, the activity of HaH steadily increased during the study period from 5,185 to 8,086 episodes per year. Supplemental Material - Table 1S depicts yearly HaH activity for each individual hospital.

Table 1 summarizes the main characteristics of the patients included in HaH, distinguishing among three relevant timeframes, covering the patient’s baseline characteristics before the admission, the hospitalization episode, and the health outcomes assessed at 30-days post-discharge. The average age of HaH patients was at 73 years, with a slightly higher representation of women. A substantial proportion of HaH episodes corresponded to high-risk patients (i.e., with AMG score above the 95th percentile of the AMG distribution for the entire population of Catalonia). The study group showed a substantial prevalence of health-related social needs associated with housing and economic conditions. Overall, HaH had high use of healthcare resources during the year before the acute episode.

**Table 1:** Patient’s clinical characteristics and outcomes of the intervention of all patients admitted to HaH

	**HaH = 31,901**
**Demographics & baseline use of resources**	
**Age, mean (sd)**^a^	73.11 (16.73)
**Gender; n (%)**^a^	
**Male**	15,214 (47.69)
**Female**	16,687 (52.31)
**AMG, mean (sd)**^a^	29.51 (16.48)
** AMG category, n (%)**	
***Very low risk*** ***< P***_***50***_	266 (0.83)
***Low risk [P***_***50***_-***P***_***80***_***)***	1,769 (5.55)
***Moderate risk [P***_***80***_-***P***_***95***_***)***	4,723 (14.81)
***High risk [P***_***95***_-***P***_***99***_***)***	5,476 (17.17)
***Very high risk ≥*** ***P***_***99***_	19,667 (61.64)
**Patients with HRSN associated to housing and economic conditions, n (%)**^a^	5,063 (15.86)
**Patients with HRSN associated to family and social environment, n (%)**^a^	9,903 (31.03)
**Patients receiving palliative care, n (%)**	1,437 (4.5)
**Patients requiring hospital admissions within the 12 months before the admission, n (%)**^a^	15,957 (50.16)
**Patients requiring emergency room visits within the 12 months before the admission, n (%)**^a^	25,812 (81.14)
**Total Expenditure in € within the 12 months before the admission, median (P**_**25**_-P_**75**_**)**^a^	4,153.4 (1,695.8 - 8424.6)
**Hospitalization episode**	
**LoS, mean (sd)**	8.47 (6.34)
**Patients requiring in-hospital all-cause readmissions, n (%)**	1,706 (5.35)
**Patients requiring emergency room visits without in-hospital readmission, n (%)**	1,339 (4.20)
**Mortality, n (%)**	103 (0.32)
**Queralt Index, mean (sd)**	28.34 (16.24)
**Case Mix Index**	0.66
**Health outcomes 30 days after discharge**	
**Mortality, n (%)**	1,383 (4.35)
**Patients requiring hospital admissions, n (%)**	3,327 (10.42)
**Patients requiring emergency room visits, n (%)**	6,136 (19.29)
**Total Expenditure in €, median (P**_**25**_-P_**75**_**)**	279.1 (119.3 - 758.7)

*AMG* stands for Adjusted Morbidity Groups, *HRSN* for health-related social needs and *LoS* for length of stay

^a^Matching variables

**Fig. 2:**
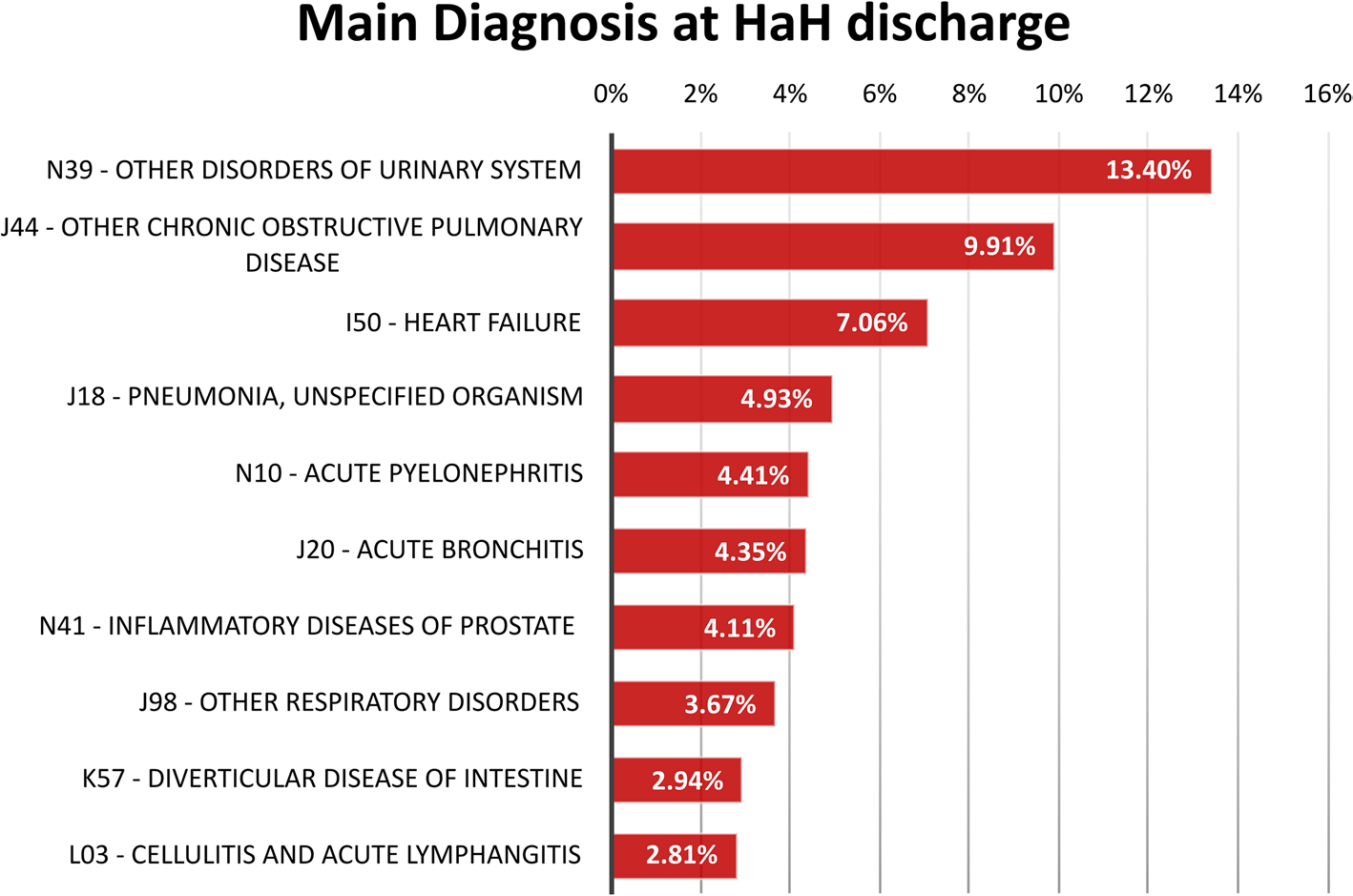
Top 10 most prevalent main diagnoses at discharge in patients admitted to HaH

The acute episode showed low mortality rates in HaH and moderate levels of complexity, measured using the Queralt index and CMI. HaH was interrupted in 1,706 (5.35%) cases, with patients requiring re-admission to in-hospital settings. Moreover, 1,339 (4.20%) patients visited the emergency room without the need to discontinue the HaH. The ten leading main diagnoses at discharge in HaH are depicted in Fig. 2.

### Comparisons between hospital at home and conventional hospitalizations

Table 2 compares the characteristics of HaH with its matched control group of patients under conventional hospitalization. Mortality during the acute episode was low and similar between intervention and controls. The effect size analyses indicated that the severity of the acute episodes, measured using the Queralt index and the CMI, was significantly higher in conventional hospitalizations than in HaH. Also, the LoS was significantly longer in HaH than in conventional hospitalizations. Despite the statistical significance, the differences observed in all endpoints between HaH and conventional hospitalization were associated with a small effect size (i.e., the differences between groups were 0.2 to 0.5 times the SD).

**Table 2:** Comparison of patients’ clinical characteristics and the outcomes of the intervention between HaH and controls

	Matched HaH	Matched control	Effect size (CI)
	*n* = 24,802	*n* = 24,802	
**Demographics & baseline use of resources**			
**Age, mean (sd)**	73.15 (16.31)	72.73 (16.29)	-0.03 [-0.04, -0.01]
**Gender; n (%)**			
***Male***	11,644 (46.95)	11,984 (48.32)	0.01 [0.01, 0.02]
***Female***	13,158 (53.05)	12,818 (51.68)	
**AMG, mean (sd)**	28.36 (15.81)	27.87 (15.82)	-0.03 [-0.05, -0.01]
**Patients with HRSN associated to housing and economic conditions, n (%)**	2,986 (12.04)	2,935 (11.83)	-0.01 [-0.01, 0.02]
**Patients with HRSN associated to family and social environment, n (%)**	7,229 (29.15)	7,072 (28.51)	-0.01 [-0.01, -0.03]
**Patients receiving palliative care, n (%)**	937 (3.78)	621 (2.5)	-0.71 [-0.06, -0.09]
**Patients requiring hospital admissions within the 12 months before the admission, n (%)**	11,580 (46.83)	11,050 (44.75)	-0.01 [-0.01, 0.03]
**Patients requiring emergency room visits within the 12 months before the admission, n (%)**	19,611 (79.31)	18,427 (74.63)	-0.02 [-0.04, 0.00]
**Total Expenditure in € within the 12 months before the admission, median (P**_**25**_**-P**_**75**_**)**	3,697.46 (1,522.40 – 7422.74)	3,399.38 (1380.34 – 7163.79)	-0.01 [-0.01, -0.03]
**Hospitalization episode**			
**LoS, mean (sd)**	8.46 (6.05)	7.09 (5.83)	-0.23 [-0.25, -0.21]
**Patients requiring in-hospital all-cause re-admissions, n (%)**	1,204 (4.85)	N.A	N.A
**Patients requiring emergency room visits without in-hospital readmission, n (%)**	923 (3.72)	N.A	N.A
**Mortality, n (%)**	76 (0.31)	112 (0.45)	0.01 [0.01, 0.02]
**Queralt Index, mean (sd)**	28.04 (15.55)	36.69 (21.96)	0.45 [0.40, 0.53]
**Case Mix Index**	0.65	0.74	0.32 [0.31, 0.34]
**Health outcomes 30 days after discharge**			
**Mortality, n (%)**	973 (3.94)	1112 (4.5)	0.01 [0.01, 0.02]
**Patients requiring hospital admissions, n (%)**	2,003 (8.08)	1,625 (6.58)	-0.07 [-0.09, -0.06]
**Patients requiring emergency room visits, n (%)**	4,109 (16.62)	3,968 (16.07)	-0.01 [-0.03, 0.01]
**Total Expenditure in €, median (P**_**25**_**-P**_**75**_**)**	809.92 (344.65 – 2,276.98)	681.11 (285.89 – 1,786.66)	-0.03 [-0.05, -0.02]
**Rubin’s B**	0.003		
**Rubin’s R**	1.001		

*AMG* stands for Adjusted Morbidity Groups, *HRSN* for health-related social needs and *LoS* for length of stay. The comparability of the matched groups is assessed by Rubin’s B and Rubin’s R, considered acceptable if Rubin’s B is less than 0.25 and Rubin’s R is between 0.5 and 2

During the 30 days after discharge, mortality rates were low, with no differences between the intervention and the control group. Likewise, re-admissions, visits to the emergency room, and healthcare expenditure were also similar between HaH and controls.

### Heterogeneities among hospitals

Comparisons among the 27 hospitals showed huge heterogeneities in HaH, in several dimensions, including age at admission (hospital mean values ranging from 62.16 to 83.39 years), multimorbidity-complexity within the 12-month period before admission expressed by AMG scoring (from 19.47 to 38.79), and severity of the acute episode assessed either using the APR-DRG (from 0.54 to 0.87) or the Queralt index (from 13.34 to 42.31). Likewise, similar inter-hospital variability was also observed in all other two variables analyzed: LoS (from 4.8 to 14.7 days) and percent of repeaters, indicating patients with more than one HaH episode during the study period (from 8.8% to 33.6%).

The cluster analysis grouped hospitals into four clusters based on comparable patient characteristics and outcomes. Cluster 1 comprised the seven community hospitals and treated patients slightly older than the other hospitals, 76.20 (4.08). The baseline morbidity burden, AMG score of 29.83 (3.08), and complexity of the episodes, CMI of 0.65 (0.04), were on average, and the LoS was the lowest, 7.96 (3.20) days.

Cluster 2, composed of five high-tech hospitals and one general hospital, had patients with similar ages to Cluster 1, 75.67 (5.01) years, but showed highest morbidity burden, AMG of 34.38 (4.07), the highest severity of the acute episodes, CMI of 0.7 (0.03) and the second longest LoS, 9.8 (2) days.

Cluster 3, integrated by nine general hospitals, exhibited values close to average across indices [Age: 71.51 (3.42) years, AMG: 29.14 (3.05), CMI: 0.69 (0.08), LoS: 8.28 (1.5) days] and showed the lowest patient reiteration rate, 12.03% (7.54).

Cluster 4 included a mix of three high-tech and one general hospital and treated the youngest patients, 65.21 (3.3) years, with the lowest morbidity burden, AMG score of 22.05 (1.91), and the lowest acute episode complexity, CMI of 0.6 (0.05), but experienced the longest LoS, 9.96 (2.71) days. Further details are available in the Supplemental Material.

### Qualitative assessments and expert recommendations

The full set of results of the quantitative analyses (Supplemental Material- Figs. 1S-8S and Table 2S-4S) were presented to the panel of experts for discussion and interpretation.

Overall, the experts agreed that HaH is safe and provides value to the healthcare system, with similar health outcomes than conventional hospitalization and, based on their own perception, has positive impacts on patients’ and professionals’ experience. The latter assertion was supported by empirical findings from previous assessments of a high-tech hospital with early adoption of HaH services within the Catalan health system [8, 34] and evidence documented in the regional consensus document [13]. HaH may also result in savings associated with fewer personnel and structure requirements [4, 42]. Nevertheless, there was consensus regarding the limitations of case-mix tools currently used (e.g., APR-DRG) to fully reflect the care needs (and, therefore, actual costs) of HaH patients. The experts agreed that new and more accurate case-mix tools should be developed, and studies based on analytical accounting should be conducted to appropriately quantify economic impact of HaH.

The experts agreed that heterogeneity in patient profile and outcomes was expected and identified three important sources of this heterogeneity: i) maturity of HaH teams (i.e., mature teams tend to admit older and more complex patients), ii) hospital strategies to use HaH in a sub-set of patients with specific diagnoses, and iii) local ecosystem (e.g., lack or availability of certain integrated care services in the area).

**Fig. 3:**
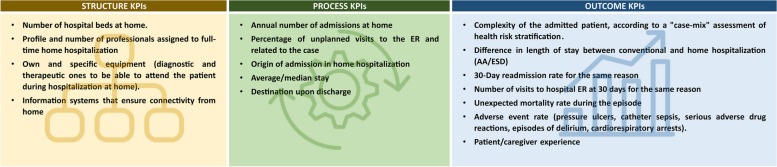
List of proposed KPIs selected from the 2020 document on regional HaH standardization [13]

Considering the exhaustive list of KPIs provided in the local 2020 recommendation document and the potential heterogeneities and challenges of this service identified in the current study, the group of experts created and selected a set of 16 KPIs that are generalizable to other healthcare system environments for continuous monitoring of HaH quality (Fig. 3).

A detailed list of highlights and specific expert interventions from the two qualitative sessions is provided in the Supplementary Material.

## Discussion

The mixed-methods approach adopted in the current research contributed to enriching the interpretation, and enhancing the potential for generalization of the results, of the retrospective quantitative assessment of consolidated HaH delivery in 27 different hospitals of the same healthcare system. The outcomes observed in HaH were aligned with relevant reports [1–4, 43–47] fully supporting the healthcare value generation of HaH, as well as its potential for capacity building of hospital beds and contributions to the care continuum. Experts highlighted HaH's safety and significant value in the healthcare system, emphasizing similar health outcomes compared to conventional hospitalization and positive impacts on patients and professionals. Analytical comparisons within the 30 days post-discharge revealed low mortality rates with no significant differences between HaH and control groups. Similarly, rates of re-admissions, ER visits, and healthcare expenditure were similar, emphasizing the safety of HaH compared to traditional hospitalization.

Previous research suggested cost savings due to reduced personnel and structural requirements [33]. Additionally, recognition from the OECD designating Catalan HaH programs as a "Best Practice in Public Health" reinforces their effectiveness [48]. Scaling up HaH across Spain and other EU27 countries could lead to substantial cost savings, estimated at EUR 6.75 per person annually until 2050, contributing to 0.004% of total health expenditure [48]. The OECD report underscores HaH's role in enhancing care integration and strengthening community-based care, shaping it as a pivotal driver in healthcare system integration [48]. Overall, the study outcomes encourage further expansion of the regional adoption of HaH, following the recommendations generated by the group of experts.

The panel of experts fully agreed with the need for continuous long-term monitoring of CQI after successfully adopting the service. In Catalonia, a consensus document for monitoring HaH services identified a comprehensive list of resources needed for adequate HaH service delivery and nearly 70 KPIs suited to the local characteristics and the type of data collected by the information systems of the Catalan Health Service [13]. In the current work, the experts identified a list of 16 essential KPIs to be considered for monitoring HaH services regardless of the characteristics of the healthcare system.

One of the intriguing features of HaH is the capacity of this service to save costs. Thus, although most studies appear to support the idea that HaH saves hospitalization costs, reviews addressing this issue have warned about the low quality and potential biases associated with the assessment of this outcome [49, 50]. The experts participating in qualitative sessions reached two important conclusions in this regard. First, this question cannot be fully answered without analytic accounting approaches. Owing to the relative lack of maturity and high heterogeneity of HaH services, the reimbursement approach to cost assessment does not appropriately reflect the actual resource use. In our environment, some hospitals have adopted analytical cost assessments that allow an accurate assessment of costs [33, 34]. However, the cost assessment in most of them had to be approached from a reimbursement perspective, limiting the strength of conclusions in this regard. Second, the experts agreed that case-mix tools typically used (and generally accepted) for reimbursement purposes (e.g., DRG) are relatively well suited to reflect the care needs of individuals admitted to conventional hospitalization but fail to do so in HaH. The expert group agreed that the implementation of analytical accounting should be extended to all hospitals to build up adequate reimbursement strategies. This approach would contribute to enhancing investments in healthcare innovation that, in turn, generate efficiencies both at hospital and health system levels. Analytical accounting would also provide a rationale for specific reimbursement plans favoring hospital-profiled service delivery. Alternatively, more accurate risk stratification models recently developed [30] should be explored as tools for a complexity-driven approach to reimbursement of HaH services.

The observed heterogeneities are consistent with disparities found in the literature [2–4]. However, the assessment of multiple hospitals within the same healthcare system allowed us to investigate these differences regardless of the healthcare structure, payment model, cultural constraints and/or type of professionals involved that may vary between countries and systems. Aside from the type of hospital, the experts identified other sources of heterogeneity that may arise when deploying HaH at the healthcare system level. These sources of heterogeneity include strategic decisions at the hospital level (e.g., use HaH to boost a particular type of service without compromising the number of beds) or contextual service availability (e.g., use HaH to counteract the lack of intermediated care services in a given area). Countries willing to deploy HaH across the healthcare system should be aware of these potential heterogeneities when planning assessment and payment models.

### Study limitations

We acknowledge some intrinsic limitations of the current study, mostly related to the use of registry data without information on details of both complexities and clinical incidences during HaH episodes. Despite the application of an accurate matching strategy between intervention and control groups, such as the clinical decision triggering patient admission to HaH instead of conventional hospitalization, which was poorly registered in the records. Moreover, the lack of analytical costs was also an important constraint assessment of the potential of value generation of HaH, as well as to explore the impact of reimbursement policies on hospitals’ heterogeneities. As described above, all economic calculations in the current study were based on expenditure data [32]. However, we believe that the characteristics of the study design and the availability of clinical and analytical data from the area [33, 34] positively influenced the analyses carried out in the current research and facilitated recommendations for enhancing the quality of service delivery that can be generalized to other integrated care services.

Finally, it is worth mentioning that our heterogeneity analysis gravitated around the center perspective, identifying differences among them and exploring potential clusters of hospitals; future research shall incorporate the perspective of patient profile within this case-mix of hospital profiles.

## Conclusions

The current study confirms safety and value generation of HaH. The service efficiently reduced hospital occupation and showed high potential to foster continuity of care, which encourages further expansion of the program at regional level.

We found that HaH is delivered in a heterogeneous case-mix of healthcare scenarios that may also result in heterogeneous outcomes. Therefore, aside from general key performance indicators, hospital-profiled indicators should be established to monitor for CQI of the service after adoption.

Our analysis and highlights of the panel of experts may help policymakers to anticipate features of this service in the advent of a system-wide implementation of HaH. Likewise, the recommendations from a panel of experts provided in this study can be used as basis for planning HaH monitoring in other countries.

### Supplementary Information

**Additional file 1: Supplemental Material S1. **Five years of Hospital at Home adoption in Catalonia: impact, challenges, and proposals for quality assurance – Supplementary Material.

## Data Availability

The datasets used and/or analyzed during the current study are available from the corresponding author upon reasonable request.

## Abbreviations

AMG: Adjusted Morbidity Groups
APR –DRG: All-Patient Refined Diagnosis Related Groups
CHSS: Catalan Health Surveillance System
CMI: Case Mix Index
CQI: Continuous Quality Improvement
LoS: Length of Stay
HaH: Hospital at Home
KPI: Key Performance Indicator
PSM: Propensity Score Matching
